# Structural Insights into the Human Metapneumovirus Glycoprotein Ectodomain

**DOI:** 10.1128/JVI.01726-14

**Published:** 2014-10

**Authors:** Cedric Leyrat, Guido C. Paesen, James Charleston, Max Renner, Jonathan M. Grimes

**Affiliations:** a Division of Structural Biology, Wellcome Trust Centre for Human Genetics, University of Oxford, Oxford, United Kingdom; b Diamond Light Source Ltd., Diamond House, Harwell Science and Innovation Campus, Didcot, Oxfordshire, United Kingdom

## Abstract

Human metapneumovirus is a major cause of respiratory tract infections worldwide. Previous reports have shown that the viral attachment glycoprotein (G) modulates innate and adaptive immune responses, leading to incomplete immunity and promoting reinfection. Using bioinformatics analyses, static light scattering, and small-angle X-ray scattering, we show that the extracellular region of G behaves as a heavily glycosylated, intrinsically disordered polymer. We discuss potential implications of these findings for the modulation of immune responses by G.

## TEXT

Human metapneumovirus (HMPV) is a ubiquitous pathogen of the Pneumovirinae subfamily of the Paramyxoviridae and causes serious respiratory illness in infants, young children, the elderly, and immunocompromised individuals (1–4). HMPV has a negative-sense, nonsegmented, single-stranded RNA genome of approximately 13 kb that encodes 9 proteins. Three of these are membrane-anchored glycoproteins, namely, the attachment (G), the small hydrophobic (SH), and the fusion (F) proteins. The F protein mediates fusion of the viral and cellular membranes during viral entry, induces syncytium formation in infected cells, and determines the cellular host range (5–8). While F is highly conserved, is immunogenic, and induces protective antibodies (9–11), the other surface glycoproteins, G and SH, have been shown to be only weakly immunogenic (11–14).

The G protein has been associated with binding to cellular glycosaminoglycans (15); however, this function was shown to be strain dependent (16). G-deleted recombinant HMPV is attenuated and induces high titers of HMPV-neutralizing serum antibodies, which confer protection against wild-type HMPV (14). Immunological studies have suggested that G inhibits host cell innate immune responses by targeting RIG-I-dependent gene transcription (17) and Toll-like receptor 4 (TLR-4)-dependent signaling (18, 19), although some of these findings have been mitigated (20, 21). Recently, deletion of the G and SH genes was shown to reduce HMPV internalization by monocyte-derived dendritic cells, leading to decreased activation of CD4^+^ T cells (22).

**FIG 1:**
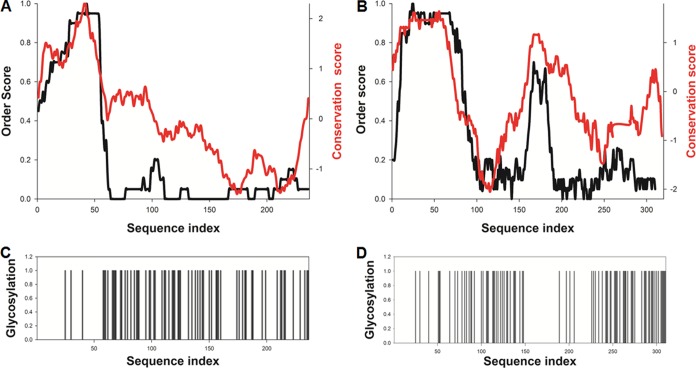
Computational analysis of HMPV (A and C) and HRSV (B and D) G sequence conservation, order/disorder propensity, and glycosylation sites. (A and B) The predicted disorder propensities and sequence conservation profiles are shown by black and red lines, respectively. Meta-disorder predictions were calculated following procedures described in reference 59. Sequence conservation was calculated using AL2CO (60) by applying a sliding average on a 20-residue window. (C and D) Location of predicted glycosylation sites along the amino acid sequence, shown as histogram bars, based on GlycoPred server output (61). HMPV sG (strain NL-1-00 [A1]) is predicted to contain 5 *N*-linked and 59 *O*-linked glycosylation sites, constituting an average of one site for every three residues.

In order to investigate the structural properties of HMPV G, we employed *in silico* predictions to compare conservation, disorder propensity, and localization of glycosylation sites in HMPV and the closely related human respiratory syncytial virus (HRSV) G (Fig. 1). Both glycoproteins possess a short conserved and structured N-terminal intracellular tail, followed by a transmembrane region (residues 31 to 53 in HMPV and residues 41 to 63 in HRSV) and an extracellular ectodomain (sG) of 182 and 236 amino acids in HMPV-A and in HRSV, respectively. The sG sequence is poorly conserved (23–26), with the exception of the cysteine-rich motif, present exclusively in HRSV (residues 160 to 190), which has been linked with immunomodulatory functions (27–29). Interestingly, the most variable regions are located near the C terminus and away from the transmembrane region (Fig. 1A and B), likely reflecting immune pressure. sG is predicted to be disordered, consistent with the large number of *O*-glycosylation sites (Fig. 1C and D) associated with a high content of serine, threonine, and proline residues (18.5, 21.7, and 10.9%, respectively).

**FIG 2:**
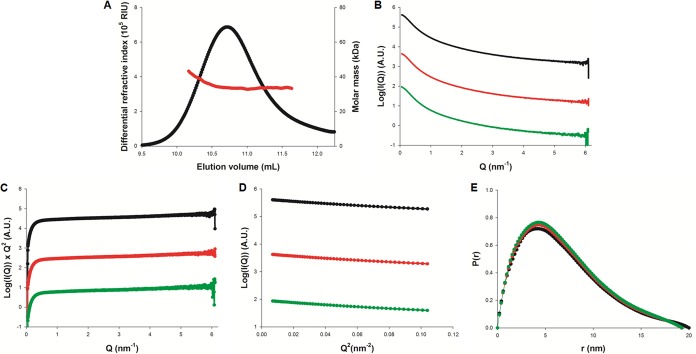
Biophysical characterization of HMPV sG. (A) Molecular mass determination of HMPV sG using SEC-MALLS-RI. The protein was purified by size exclusion chromatography on an S200 column equilibrated with 20 mM Tris (pH 7.5) and 150 mM NaCl prior to analysis. The black line shows the SEC elution profile as monitored by refractometry. The red line shows the molecular mass calculated from light scattering and refractometry data. (B, C, D, and E) Small-angle X-ray scattering (SAXS) experiments; (B) scattering curves of sG measured at concentrations of 4, 6, and 8 mg/ml are shown in green, red, and black, respectively; (C) Kratky plots showing linear behavior in the high Q range; (D) Guinier plots showing linear behavior in the low Q range; (E) distance distribution functions [P(r)] calculated using GNOM (62).

Next, sG (residues 54 to 236) from strain NL-1-00 (A1) was cloned into pHLsec with an N-terminal secretion signal and a C-terminal His tag, transiently expressed in HEK293T cells, and purified from culture medium following standard procedures (30). Characterization using size exclusion chromatography combined with multiangle laser light scattering and refractometry (SEC-MALLS-RI) (31) (Fig. 2A) indicates a molecular mass varying between 34 and 41 kDa (±1%). Comparison with the sequence-derived molecular mass (20.0 kDa) suggests that sG is a highly glycosylated monomer, with carbohydrates accounting for roughly 50% of the measured molecular mass, a property that may contribute to virion stability by preventing dehydration (32).

To gain insight into the solution structure of sG, we used small-angle X-ray scattering (SAXS) (Fig. 2B). Guinier plots were linear and unaffected by protein concentration (Fig. 2C), with a measured radius of gyration (*R_g_*) of 5.5 nm. Kratky plots were linear at high scattering vector Q (Q = 4π × sin(θ)/λ) (Fig. 2D), indicating that sG behaves as a random polymer. The pair-distance distribution function P(r) displayed a pronounced tail with a maximal intramolecular distance (*D*_max_) of 20 nm (Fig. 2E), which is characteristic for elongated or disordered proteins (33).

**FIG 3:**
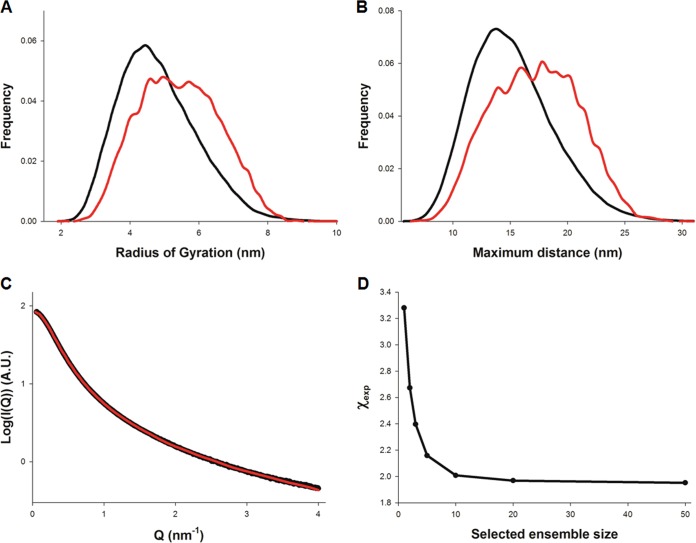
Analysis of sG flexibility using the ensemble optimization method (EOM). (A and B) Calculated radius of gyration (*R_g_*) and maximal intramolecular distance (*D*_max_) distributions of the starting (black line) and optimized ensembles (red line). (C) Fitted SAXS profile of sG measured at 8 mg/ml. The experimental data are shown in black and the theoretical scattering from the optimized ensemble in red. (D) Variation of the goodness of fit (χ_exp_) as a function of the optimized ensemble size.

We then used ensemble optimization to quantify sG flexibility. sG was modeled using an ensemble of bead models, as implemented in the program RANCH, and the data were fitted using GAJOE (34). Although these models are coarse and cannot reproduce the branched nature of the glycoprotein, they remain useful in extracting the distributions of *R_g_* and *D*_max_ (Fig. 3A and B), indicating that sG populates a broad ensemble of conformations with *R_g_* of 3 to 8 nm and *D*_max_ ranging from 10 to >25 nm. SAXS profiles were well fitted (Fig. 3C), with the goodness of fit (χ_exp_) decreasing smoothly from 3.3 to 2.0 when the optimized ensemble size was varied between 1 and 50 models (Fig. 3D), consistent with high levels of intrinsic disorder (35).

**FIG 4:**
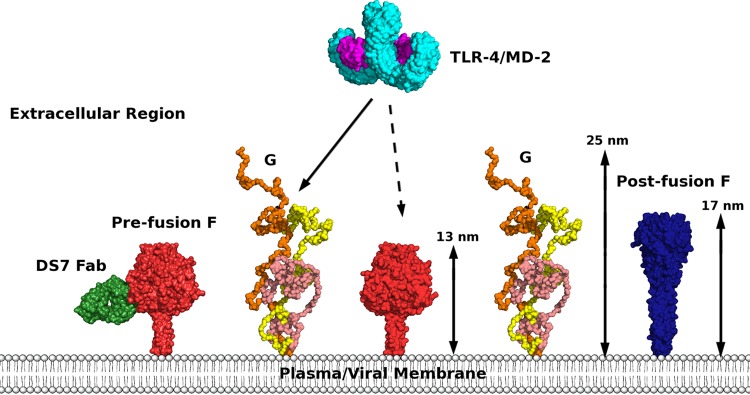
Comparison of the molecular dimensions of the extracellular regions of the HMPV F and G proteins, illustrating the G protein induced steric hindrance potentially hampering host factor-F protein interactions. The extracellular region of the Toll-like receptor 4–myeloid differentiation factor 2 (TLR-4/MD-2) complex (PDB identifier 3FXI) is represented as an example of such host factor. Homology models of the F protein trimers in the pre- and postfusion states form protrusions of 13 and 17 nm, respectively. Models were generated in HOMER (63) and are represented as red and blue surfaces. The homodimeric TLR-4 is shown in cyan and MD-2 in magenta, and three superimposed low-resolution models of sG are shown in orange, wheat color, and yellow. The anti-HMPV DS7 Fab bound to the prefusion model of F is shown in green based on PDB identifier 4DAG.

The large dimensions of sG and the reported association of F and G at the surface of viral particles (36, 37) suggested that G may have a shielding function and prompted us to compare the size of the two proteins (Fig. 4). The trimeric fusion protein F, which can exist in both pre- and postfusion conformations, possesses a large extracellular region for which extensive structural information is available (38–42). The ability of sG to extend up to >25 nm from the membrane would allow the protein to tower above the smaller F trimers, a phenomenon that might be amplified through oligomerization mediated by the transmembrane region (43). Steric hindrance by G may additionally decrease F binding to neutralizing antibodies, such as DS7 (a Fab fragment has been solved in complex with a fragment of the HMPV F protein [42]), or host factors, such as the innate immune sensor TLR-4–myeloid differentiation factor 2 (MD-2) complex, which is activated through binding of HRSV F to MD-2 (44).

This “steric masking” hypothesis is supported by the hypervariability of the C-terminal region of sG, the increased capture radius and binding rates associated with intrinsic disorder (45, 46), and the decreased binding affinity of soluble proteins to membrane-anchored substrates in the presence of crowding factors (47). In addition, because of the nonprotective and cross-protective nature of antibodies directed against G and F, respectively (10–14, 48), transient immunity leading to reinfection could be explained if G can reduce the immunological footprint associated with F. This is consistent with the increased CD4^+^ T cell activation observed with HMPV lacking the G and SH proteins (22). Interestingly, an avian metapneumovirus isolate bearing a long (585-residue) G protein was found to replicate efficiently without signs of disease in domestic turkeys, suggesting decreased activation of the innate immune response (49–51).

The properties of pneumovirus G proteins, such as intrinsic disorder, sequence hypervariability, and heavy *O*-glycosylation, contrast with the structured attachment glycoproteins in other paramyxoviruses (52, 53). Interestingly, the surface glycoprotein GP from Ebola virus is dominated by a mucin-like domain of 150 amino acids, which was shown to shield viral epitopes and impair immune recognition (54, 55). This suggests similarities between immune evasion strategies employed by pneumoviruses and filoviruses, whose evolutionary relationship was recently highlighted by structural comparison of the matrix and M2-1 transcriptional antiterminator proteins (56–58).
